# An Approach for Combining Clinical Judgment with Machine Learning to Inform Medical Decision Making: Analysis of Nonemergency Surgery Strategies for Acute Appendicitis in Patients with Multiple Long-Term Conditions

**DOI:** 10.1177/0272989X241289336

**Published:** 2024-10-23

**Authors:** S. Moler-Zapata, A. Hutchings, R. Grieve, R. Hinchliffe, N. Smart, S. R. Moonesinghe, G. Bellingan, R. Vohra, S. Moug, S. O’Neill

**Affiliations:** Department of Health Services Research and Policy, London School of Hygiene & Tropical Medicine, London, UK; Department of Health Services Research and Policy, London School of Hygiene & Tropical Medicine, London, UK; Department of Health Services Research and Policy, London School of Hygiene & Tropical Medicine, London, UK; Bristol Surgical Trials Centre, University of Bristol, Bristol, UK; College of Medicine and Health, University of Exeter, Exeter, UK; Department for Targeted Intervention, Division of Surgery and Interventional Science, University College London, NHS foundation Trust, London, UK; Department for Targeted Intervention, Division of Surgery and Interventional Science, University College London, NHS foundation Trust, London, UK; Trent Oesophago-Gastric Unit, City Campus, Nottingham University Hospitals NHS Trust, Nottingham, UK; Department of Colorectal Surgery, Royal Alexandra Hospital, Paisley, UK; Department of Health Services Research and Policy, London School of Hygiene & Tropical Medicine, London, UK

**Keywords:** machine learning, expert judgement, local instrumental variables, acute appendicitis, long-term conditions

## Abstract

**Background:**

Machine learning (ML) methods can identify complex patterns of treatment effect heterogeneity. However, before ML can help to personalize decision making, transparent approaches must be developed that draw on clinical judgment. We develop an approach that combines clinical judgment with ML to generate appropriate comparative effectiveness evidence for informing decision making.

**Methods:**

We motivate this approach in evaluating the effectiveness of nonemergency surgery (NES) strategies, such as antibiotic therapy, for people with acute appendicitis who have multiple long-term conditions (MLTCs) compared with emergency surgery (ES). Our 4-stage approach 1) draws on clinical judgment about which patient characteristics and morbidities modify the relative effectiveness of NES; 2) selects additional covariates from a high-dimensional covariate space (*P* > 500) by applying an ML approach, least absolute shrinkage and selection operator (LASSO), to large-scale administrative data (*N* = 24,312); 3) generates estimates of comparative effectiveness for relevant subgroups; and 4) presents evidence in a suitable form for decision making.

**Results:**

This approach provides useful evidence for clinically relevant subgroups. We found that overall NES strategies led to increases in the mean number of days alive and out-of-hospital compared with ES, but estimates differed across subgroups, ranging from 21.2 (95% confidence interval: 1.8 to 40.5) for patients with chronic heart failure and chronic kidney disease to −10.4 (−29.8 to 9.1) for patients with cancer and hypertension. Our interactive tool for visualizing ML output allows for findings to be customized according to the specific needs of the clinical decision maker.

**Conclusions:**

This principled approach of combining clinical judgment with an ML approach can improve trust, relevance, and usefulness of the evidence generated for clinical decision making.

**Highlights:**

- Machine learning (ML) methods have many potential applications in medical decision making, but the lack of model interpretability and usability constitutes an important barrier for the wider adoption of ML evidence in practice.
- We develop a 4-stage approach for integrating clinical judgment into the way an ML approach is used to estimate and report comparative effectiveness.
- We illustrate the approach in undertaking an evaluation of nonemergency surgery (NES) strategies for acute appendicitis in patients with multiple long-term conditions and find that NES strategies lead to better outcomes compared with emergency surgery and that the effects differ across subgroups.
- We develop an interactive tool for visualizing the results of this study that allows findings to be customized according to the user’s preferences.

The rapid recent development of machine learning (ML) algorithms has catalyzed a new paradigm for health care research,^1–3^ including on risk prediction with high-dimensional data,^4,5^ estimation of optimal combination drug therapies,^6,7^ and the use of artificial intelligence for diagnostic imaging.^8^ ML methods can search over high-dimensional genetic, clinical, and sociodemographic data from electronic health records to detect relevant treatment by subgroup interactions.^9–13^ These applications of ML have the potential to generate evidence that can inform more personalized medical decision making.

Despite the growing availability of evidence from ML, clinical practitioners and policy makers can perceive many of these methods as “black boxes,” and this may undermine the appropriate use of ML in decision making.^14^ This has been compounded by a number of studies that highlight the risk of bias in the development and use of algorithms (e.g., Obermeyer et al.^15^). Algorithms may perform poorly for some groups, for instance due to being trained on data in which these groups have low prevalence,^16^ or because historical data may reflect and reinforce previous biases in decision making or opportunities.^17^ This may be exacerbated by the use of proxy variables in place of the variable of interest, particularly where the degree of mismeasurement differs across groups.^15^ Conceptually, one can think of a model or algorithm as being “fair” if it performs equally across subgroups defined by sensitive variables (e.g., gender/sex, age).^18^ Improving the fairness of ML approaches is an active area of research (see Pessach and Shmueli^19^ for a useful review of this literature). Moreover, checklists that have been developed to assess risks of high-dimensional confounding and spurious subgroup findings from ML models can also help mitigate these concerns.^20–22^

While including clinical judgment in the design of ML models can also improve clinical trust,^23^ there remains a gap in the literature about how to integrate clinical judgment for ensuring usability, trust, and sufficient understanding of ML output by evidence users. An important area where clinical decision making may be improved by clinical investigators codeveloping an ML approach is in generating appropriate evidence to target interventions for people with multiple long-term conditions (MLTCs; or multiple comorbidities). These populations are often excluded from randomized controlled trials (RCTs), and there is a lack of evidence on how comorbidities, alone and in combination with each other, and with other risk factors (e.g., age), might influence response to interventions. This leads to challenges for clinical decision makers who have to rely solely on their clinical judgment in deciding which intervention a patient should receive.^24,25^ New approaches for integrating clinicians’ explicit and tacit knowledge^i^ about decision making for these patients into the design and translation stages of ML models are required to help target interventions. This article aims to present an approach for integrating clinical judgment into the development and reporting of ML approaches for estimating subgroup effects.

We develop a 4-stage approach for harnessing clinical judgment with ML to provide evidence to inform decision making. We exemplify the approach in evaluating nonemergency surgery (NES) strategies (including, e.g., antibiotic therapy and/or delayed surgery) versus emergency surgery (ES) for patients with acute appendicitis who have MLTCs. RCTs have reported that NES strategies may lead to similar outcomes to ES for people with acute appendicitis but have excluded older patients and underrepresented those with MLTCs.^27,28^ Some published clinical guidelines have suggested that frailty, as well as individual comorbidities, such as diabetes and heart disease,^24,29^ should be considered in deciding between NES strategies or ES, but not others. Hence, there is still limited evidence available to inform decision making about the choice of NES versus ES for patients with MLTCs admitted to hospital with acute appendicitis.

The ESORT study used large-scale administrative data to undertake effectiveness and cost-effectiveness analyses for 5 acute conditions (acute appendicitis, acute gallstone disease, diverticular disease, acute hernia, and intestinal obstruction) and found that NES strategies including antibiotic therapy, and/or later surgery, might be more beneficial than ES for older patients and those with severe levels of frailty (see Hutchings et al.,^30^ Moler-Zapata et al.,^31^ and Grieve et al.^32^ for further details). However, this study has not provided evidence that can inform decision making for people with MLTCs. These patients tend to be older and frailer, and these characteristics may interact with a myriad of LTCs. Hence, providing useful evidence for clinical decision making requires consideration of hundreds of variables.

We present an approach that combines expert judgment from clinical co-investigators with the use of least absolute shrinkage and selection operator (LASSO), a data-driven ML approach to covariate selection.^36^ Six clinical decision makers helped design (stage 1) and report findings (stage 4) of the study in a way that helped the ML approach estimate treatment effects for subgroups of decision-making relevance and ensure that results were clearly presented and the limitations and uncertainties discussed. This approach can be adapted to other decision-making settings, which may warrant alternative ML models or more formal approaches to expert opinion elicitation. We illustrate the approach in extending the ESORT study to undertake a new evaluation of NES strategies for people with acute appendicitis who have MLTCs.^30–32^ We develop an interactive tool that can help customize the ML output for the needs of the particular decision maker.

## Overview of the Relevant Features of the ESORT Study, the Decision Problem, and Data

### Population of Interest

We focus on acute appendicitis as this is a common reason for emergency admission. For people who have MLTCs, a vital decision is whether to have antibiotic therapy with the possibility of later surgery (NES) rather than ES. As the patient is admitted as an emergency, this decision is usually taken promptly by the clinical team, including surgeons and anesthetists. The population under consideration all had MLTCs and were identified from the Hospital Episode Statistics (HES) database.^34^ The study population met the general ESORT inclusion criteria: patients were aged 18 y or older; admitted as an emergency via an accident and emergency department or primary care referral to 1 of 175 NHS hospitals in England from April 1, 2010, to December 31, 2019; had the relevant ICD-10 diagnostic codes for acute appendicitis; and met other inclusion criteria (*N* = 24,312) (see Hutchings et al.,^30^ Moler-Zapata et al.,^31^ and Grieve et al.^32^ for further details). Here, MLTC was defined as 2 or more of 28 comorbidities at or up to 2 y prior to admission.^35^

### Comparator Strategies

Admissions were defined as receiving the ES strategy if, according to Office of Population Censuses and Surveys codes, they had a relevant operative procedure within the 7 d of eligibility (see Grieve et al.^32^ for further details). All other eligible admissions were designated as receiving NES strategies including antibiotic therapy and/or surgery after 7 d.

### Covariates

Baseline covariates were extracted from HES and included age, sex, ethnicity, and the Index of Multiple Deprivation (IMD). An indicator of frailty was derived from the Secondary Care Administrative Records Frailty (SCARF) index after excluding medical comorbidities that were in the list of 28 used to define MLTCs.^36^ Table 1 lists the baseline characteristics of the population of interest, of whom 15% had NES strategies and 85% had ES. The 28 individual comorbidities were extracted using ICD-10 codes based on the study by Stokes et al.^35^ of MLTCs in acute admissions in HES (see Table 2).

**Table 1:** Baseline Characteristics of Patients in the Cohort

Patient Characteristics, *n* (%)	Emergency Surgery (*n* = 20,669)	Nonemergency Surgery (*n* = 3,643)
Age, y		
<60	11,337 (55)	1,195 (33)
60–74	6,089 (29)	1,151 (32)
>75	3,243 (16)	1,297 (36)
Frailty		
Not frail	18,292 (88)	3,132 (86)
Frail	2,377 (12)	511 (14)
Sex		
Male	9,951 (48)	1,766 (48)
Female	10,718 (52)	1,877 (52)
Admission method		
Accident and emergency department	14,964 (72)	2,573 (71)
General practitioner referral	5,705 (28)	1,070 (29)
Ethnicity		
Not stated/unknown	1,425 (7)	178 (5)
White	17,630 (85)	3,149 (86)
Black/Black mixed	392 (2)	91 (2)
Asian/Asian mixed	833 (4)	171 (5)
Chinese/other	389 (2)	54 (1)
Index of Multiple Deprivation		
Quintile 1: most deprived	3,689 (18)	633 (17)
Quintile 2	4,010 (19)	725(20)
Quintile 3	4,151 (20)	710 (19)
Quintile 4	4,231 (20)	728 (20)
Quintile 5: least deprived	4,363 (21)	810 (22)

**Table 2:** Covariates Selected Using Clinical Judgment in Stage 1

Comorbidity	Sample Size (% NES)	Selected	Met the Volume Threshold
Alcohol misuse	1,847 (11)	No	—
Asthma	6,569 (11)	No	—
Atrial fibrillation	170 (24)	No	—
Cancer	734 (25)	Yes	Yes
Chronic heart failure	1,104 (34)	Yes	Yes
Chronic kidney disease	3,665 (23)	Yes	Yes
Chronic pain	2,725 (14)	No	—
Chronic pulmonary disease	2,948 (21)	Yes	Yes
Chronic viral hepatitis B	194 (28)	No	—
Cirrhosis	938 (8)	Yes	Yes
Dementia	405 (42)	Yes	Yes
Depression	5,213 (10)	No	—
Diabetes	6,383 (19)	Yes	Yes
Epilepsy	1,042 (12)	No	—
Hypertension	13,046 (17)	No	—
Hypothyroidism	3,369 (13)	No	—
Inflammatory bowel disease	702 (16)	Yes	Yes
Irritable bowel syndrome	1 (0)	No	—
Multiple sclerosis	200 (9)	No	—
Myocardial infarction	74 (12)	No	—
Parkinson’s disease	170 (25)	No	—
Peptic ulcer disease	142 (20)	No	—
Peripheral vascular disease	2,168 (17)	No	—
Psoriasis	381 (12)	No	—
Rheumatoid arthritis	1,065 (17)	No	—
Schizophrenia	300 (13)	No	—
Severe constipation	2,559 (13)	No	—
Stroke	42 (14)	Yes	No

NES, nonemergency surgery.

### Outcomes

The outcome measure was the number of days alive and out of hospital (DAOH) at 90 d measured from the index date.^37,38^ The calculation of DAOH used HES data on the total duration of hospitalization over the 90-d period including readmissions and the date of death from linkage to the Office for National Statistics (ONS) death record. Patients who died within the 90-d period were assigned zero DAOH.

### Overview of Analysis of Comparative Effectiveness

The main interest for decision making was the comparative effectiveness of NES strategies versus ES on the mean DAOH at 90 d for subgroups defined by baseline patient characteristics and individual LTCs. The analytical approach recognized that some prognostic variables available to the clinicians that may influence the choice of NES versus ES, such as the patient’s physiological status, were not recorded in the data. We therefore followed the original ESORT study in using an instrumental variable (IV) analysis. The IV was the hospital’s “tendency to operate,” defined as the proportion of eligible patients who had NES rather than ES in the year prior to the specific admission.

The rationale for the IV approach is that, provided the requisite assumptions for the IV to be valid are met, it can reduce the risk of bias in the estimate of relative effectiveness that is due to confounding.^39^ In particular, a major concern when attempting to estimate comparative effectiveness from observational data is that there may be unmeasured baseline prognostic differences between the comparison groups, such as the severity of the disease, which could act as confounding factors. The relative advantage of an IV approach is that it can balance these unobserved prognostic differences between the comparison groups and hence reduce the risk of bias in the estimates of treatment effectiveness.^39–41^

Our previous articles have explained in detail why the hospitals’ tendency to operate (TTO) may be a valid IV for the choice of NES versus ES,^30–32^ and have set out why the requisite underlying assumptions are plausible in this context. In brief, the hospital’s TTO needs to satisfy the following assumptions: 1) it is not correlated with the outcome except through the treatment assignment, conditional on the covariates; 2) it is strongly correlated with treatment assignment; and 3) it does not increase the probability of treatment assignment at a specific value of the IV but decreases it at higher values. A series of study design features supported the validity of the IV, including: 1) given the acute nature of the condition, it is unlikely that patients would choose to attend hospitals according to their TTO; 2) referrals from tertiary hospitals were excluded; and 3) models were adjusted for proxies for hospital quality-of-care indicators.^30–32^ Also, some tests carried out provided further reassurance, showing the IV to be strongly correlated with treatment assignment (F > 100) and to achieve good balance in observed covariates, which might suggest that it also balances the unobserved ones (see Moler-Zapata et al.^31^ for further detail).

Given that it is plausible that the TTO is a valid IV, the subsequent estimation of comparative effectiveness can mitigate the risks of bias from unmeasured confounding. A further advantage of the IV approach that we take is that it recognizes the potential for effect modification according to unobserved covariates (see also section 2 in the supplementary materials).^42,43^ The extension in this article is that within the framework of using the IV to estimate comparative effectiveness, we take a 4-stage approach to combining clinical judgment with ML to estimate comparative effectiveness for specific subgroups of decision-making relevance.

## A 4-Stage Approach for Developing Appropriate Evidence to Target the Choice of Intervention

**Figure 1:**
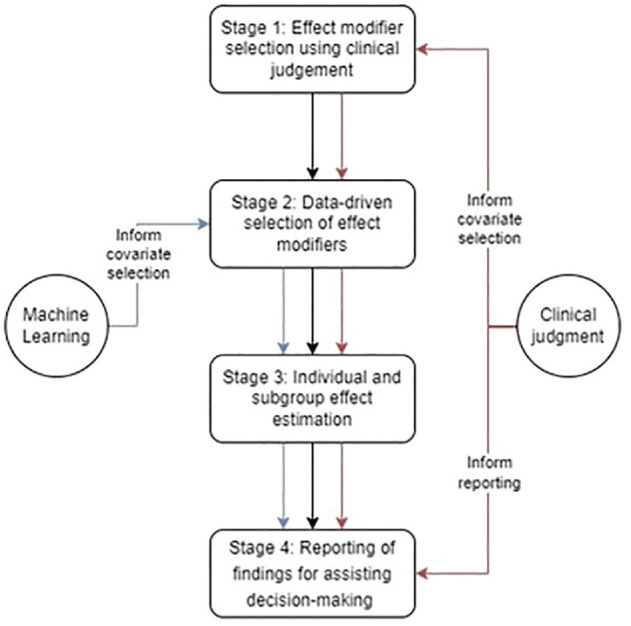
Illustration of the 4-stage approach for integrating clinical judgment into a machine learning model. Arrows connecting the rectangle show the sequence of stages. Arrows connecting the circles with the rectangles indicate the use of machine learning methods or clinical input in that stage. Color key: black, logical ordering; blue, machine learning; red, clinical judgment.

We now outline the 4-stage approach to combining clinical judgment with ML (see Figure 1). We exemplify each stage in evaluating NES versus ES for people with acute appendicitis who have MLTCs. In this approach, we draw on coproduction between investigators with a primarily methodological or primarily clinical background.^44^ Clinical judgment is provided by 6 clinical investigators (4 surgeons, 1 intensivist, and 1 anesthetist, all with at least 25 y of clinical experience) with roles as both researchers and users of research evidence. All are coauthors and were involved in the design and conduct of the study.

### Stage 1: Effect Modifier Selection Using Clinical Judgment

In preparation for stage 2, and to address the concern that ML methods may identify spurious subgroup effects at the expense of those of decision-making relevance, the clinical co-investigators used their expert judgment to identify which of the 28 comorbidities should be selected as “forced” covariates and considered as effect modifiers. The unselected comorbidities are considered in stage 2.

#### Implementation of stage 1

We undertook a survey among the clinical investigators to rate the 28 comorbidities as “low,”“medium,” or “high” priority for inclusion as forced covariates in terms of their influence on the choice of NES strategies or ES and/or their potential for modifying the relative effectiveness of ES versus NES strategies. We discussed the purpose of the exercise at an online investigators’ meeting and developed a brief survey questionnaire. Our meeting included a discussion of the challenges of using an ML approach to identify those patient characteristics that modify the effectiveness of NES strategies versus ES for patients with MLTCs. The questionnaire was circulated for comment and then modified for face validity before we circulated it to the clinical investigators. All 6 clinical investigators independently completed the survey. We designated the comorbidities with a mean rank across the responses of at least “medium” priority as forced covariates that must be included in the model and considered as effect modifiers. Nine of the 28 comorbidities were selected. Interrater agreement between the 6 clinical investigators was fair to good (intraclass correlation 0.62).

### Stage 2: Data-Driven Effect Modifier Selection

While clinical input allows us to identify suspected effect modifiers, it cannot detect those covariates that are unknown modifiers either alone or in combination with other morbidities or other covariates. One could consider a full set of treatment by covariate interactions. However, this may lead to a high-dimensional, complex model with many parameters that is overfitted to the data and yields unstable estimates. In extreme cases, estimation may be infeasible. A wide range of approaches to covariate selection are available (see chapter 3 in Hastie et al.^45^), including backwards selection, whereby variables are dropped sequentially based on their statistical significance. However, backwards selection suffers from overfitting to the data used to assess statistical significance.^46^

Here, we considered the LASSO approach to reduce the risk of overfitting by reducing model complexity through selecting variables for inclusion, thereby reducing the number of parameters estimated (see section 1 in the supplementary materials).^36,47^ LASSO can lead to regularization bias since, as the estimated coefficients are shrunk toward zero, some meaningful variables for clinical decision making may be excluded. We use a post-double selection (PDS) approach to account for regularization bias while selecting potential confounders for inclusion. PDS identifies potential confounders by estimating auxiliary regressions of 1) the treatment on covariates and 2) the outcome on covariates. The final models include the union of variables that were selected as influencing treatment or outcomes.^48,49^ This final model provides asymptotically valid standard errors for the treatment effect. Variables that are not selected in any of the auxiliary regressions are deemed not to be sufficiently important confounders to warrant inclusion. We adapt the PDS approach to account for effect modification as described below.

#### Implementation of stage 2

The covariates under consideration were from 4 broad groups:

a. Patient characteristics (age category, sex, frailty),
b. medium-/high-priority comorbidities,
c. low-priority comorbidities, and
d. covariate interactions, among comorbidities (b and c) and each comorbidity with age category, sex, and frailty (a).

Computational time increases with the number of variables considered, and so covariates and covariate interactions were dropped if any subgroup failed to meet a minimum volume threshold of 50 patients in each group (see Table S1 in the supplementary materials for a full list of covariates).^50^

Following the results of stage 1, we implement our variable selection approach as follows. Rigorous LASSO was allowed to select: (i) variables from groups (c-d) for the outcome model, while the inclusion of variables in (a-b) and the treatment indicator was “forced”, ( $X_{\mathrm{Selecte}d_{Y}}$ ),^ii^ and any variables from any of the groups (a–d) for the treatment model, while the inclusion of the instrument was “forced”, ( $X_{\mathrm{Selecte}d_{D}}$ ) (see Supplementary Table S2 for alternative approaches to selection of covariates). In the outcome model, we also include interactions between the treatment and each variable in groups (a–d) to allow for effect modification by these variables, and we use rigorous LASSO to select those to retain. We then form the union of the selected covariates and those selected based on clinical input in Stage 1. This represents the final set of effect modifiers for the subsequent estimation of subgroup effects ( $X_{\mathrm{Selected}}$ ). In summary, this step finds covariates that are included in the final models, as their omission would lead to a large omitted variable bias.

#### Stage 3: Individual and Subgroup Effect Estimation

To provide nuanced treatment effect estimates to inform more personalized decision making, we require treatment effects to be estimated at the individual level, which can then be aggregated to appropriate subgroups according to the variables previously selected by clinical judgment (stage 1) and LASSO (stage 2). As summarized in section ‘Overview of Analysis of Comparative Effectiveness’, the ESORT study used the TTO as the IV to address unobserved confounding due to the lack of baseline information on prognostic factors such as the patients’ physiology. However, as these variables could also modify the treatment response, we applied a local instrumental variable (LIV) estimator,^52–54^ as this provides consistent individual-level effect estimates in the presence of heterogeneity according to observed and unobserved confounders. These individual effects can then be aggregated to obtain estimates for the overall population and relevant subgroups (see section 2 in the supplementary materials and Basu^54^ and Moler-Zapata et al.^30^ for further details).

The intuition behind this method is that, from the data, it is possible to identify marginal patients who are in equipoise with respect to the treatment assignment decision given their level of measured and unmeasured covariates and for whom a small (marginal) change in the IV is sufficient to nudge them into the treatment group.^55^ Contrasting the outcomes for patients who are identical in measured and unmeasured covariates but have marginally different levels of the IV, we can identify marginal treatment effects (MTEs). Our LIV estimator uses the variables selected in stages 1 and 2 (see section 2 in the supplementary materials) for estimating MTEs. This approach has advantages over methods such as using fully interacted regression models with recycled predictions, which assume that there are no unobserved confounders or effect modifiers.^56,57^

In addition to variables in $X_{\mathrm{Selected}}$ and $X_{\mathrm{Forced}}$ , models for estimating treatment effects are adjusted for the patient’s ethnicity (according to categories considered in HES), IMD,^iii^ age and age-squared terms, time period, as well as teaching hospital status and hospital quality-of-care indicators (according to rates of 90-d all-cause mortality and readmissions in preceding periods). Once the individual-level treatment effects have been estimated, several approaches can be taken to identify subgroups of interest. These subgroups could be prespecified, for instance, according to the list derived in stage 1 and stage 2, or determined data adaptively.^10,11,59–63^ Here, individual-level effects are aggregated for subgroups defined by the covariates and covariate interactions selected in stage 1 and stage 2.

#### Implementation for stage 3

We estimate individual-level treatment effects by including the variables selected in stage 1 and stage 2 (see section 2 in the supplementary materials for further details). We aggregate the resultant individual-level effect estimates to obtain a subgroup effect estimate for each group defined by variables in the set of treatment effect modifiers identified in stage 1 and stage 2 (i.e., $X_{\mathrm{Selected}}\;\mathrm{and}\;X_{\mathrm{Forced}}$ ). Standard errors were obtained by nonparametric bootstrap.

#### Results for stages 1 to 3

The results for stage 1 are given in Table 2. Briefly, 7 comorbidities were chosen as being of clinical relevance; that is, they were deemed to influence 1) the choice of NES strategies versus ES, 2) the relative effectiveness of NES versus ES (i.e., effect modifiers), and 3) met the requirement that there were at least 50 observations for each comparator group at each level of the subgroup (see Table 2).

In stage 2, of the potential modifiers considered, the LASSO selected only 1 additional covariate, chronic viral hepatitis B, in the treatment assignment and outcome models (see columns 2 and 3 in Table 3). However, LASSO chose several additional covariate interactions as effect modifiers (see columns 4 and 5 in Table 3).

**Table 3:** Covariates Selected Using Post–Double Selection with LASSO in Stage 2

Comorbidity	Selected as Main Effect		Selected as Interaction	
	In Treatment Model	In Outcome Model	In Treatment Model	In Outcome Model
Age 60-74 y	—	—	Yes	Yes
Age >75 y	—	—	Yes	Yes
Frail	—	—	No	No
Female	—	—	Yes	No
Cancer	—	—	No	Yes
Chronic heart failure	—	—	Yes	Yes
Chronic kidney disease	—	—	Yes	Yes
Chronic pulmonary disease	—	—	Yes	No
Cirrhosis	—	—	Yes	No
Dementia	—	—	No	No
Diabetes	—	—	Yes	No
Inflammatory bowel disease	—	—	No	No
Alcohol misuse	No	No	No	No
Asthma	No	No	No	No
Atrial fibrillation	No	No	No	No
Chronic pain	No	No	No	No
Chronic viral hepatitis B	Yes	Yes	No	No
Depression	No	No	No	No
Epilepsy	No	No	No	No
Hypertension	No	No	No	Yes
Hypothyroidism	No	No	No	No
Multiple sclerosis	No	No	No	No
Parkinson’s disease	No	No	No	No
Peptic ulcer disease	No	No	No	No
Peripheral vascular disease	No	No	No	Yes
Psoriasis	No	No	No	No
Rheumatoid arthritis	No	No	No	No
Schizophrenia	No	No	No	No
Severe constipation	No	No	No	No
Stroke	No	No	No	No

All variables selected had to meet a minimum volume threshold of 50 for each comparator. In addition to the covariates listed in this table, some variables that were not considered as effect modifiers in stage 1 or 2 were included in the models for estimating treatment effects. These included the patient’s ethnicity, Index of Multiple Deprivation, as well as teaching hospital status and hospital acute care indicators. LASSO, least absolute shrinkage and selection operator.

Once the individual-level effects were aggregated to the overall MLTC population with acute appendicitis (stage 3), NES strategies led to, on average, 4.6 (95% confidence interval [CI]: 2.7 to 7.0) additional DAOH compared with ES. For patients older than 75 y and those with frailty, NES led to relatively greater gains in DAOH compared with ES. Figure 1 also shows that as compared with ES, NES strategies lead to gains in DAOH for most subgroups identified according to the covariates selected in stage 1 and stage 2 but with some differences in the magnitude of the gains from 21.2 (1.8 to 40.5) for patients with chronic heart failure and chronic kidney disease, to −10.4 (−29.8 to 9.1) for patients with cancer and hypertension, albeit with large uncertainty around some of the point estimates (see Figure 1).

### Stage 4: Reporting of Findings for Assisting Decision Making

In studies using ML to detect effect modifiers, the number of potential subgroup effects, and hence the complexity of the reporting, increases as the covariate space grows. Here, we identified 48 subgroups as being of policy relevance from more than 500 candidates. We recognized that the way results are presented to evidence users influences data interpretation and subsequent adoption of evidence in decision making.^64–66^ We therefore discussed with clinical co-investigators how to adapt the presentation of findings to help inform decision making. In this stage, we describe how clinical judgment was used to inform graphical presentation of the results from stages 1 to 3.

#### Implementation of stage 4

We prepared a pilot version of the interactive tool (https://github.com/silviamoler) to illustrate alternative ways of presenting results. To obtain the views of the clinical investigators on the way results can be presented, we held 6 online meetings between, at least, 2 of 3 nonclinical investigators and each clinical investigator. Meetings lasted 30 to 60 min and followed a broad topic guide covering usefulness, readability, and ease of understanding of the results to potentially inform clinical decision making. The meetings were informal, rather than interviews, given the familiarity and trust among participants, and clinical investigators were able to ask and respond to questions in the discussion. The nonclinical investigators circulated brief notes following meetings and prepared a summary of findings from the meetings for review by all investigators. We did not use formal elicitation methods as the goal of these discussions was to illustrate alternative options for reporting results and how dynamic, interactive approaches for reporting may be most helpful for bridging clinicians’ tacit with ML-generated knowledge and fostering adoption of ML evidence in practice. The results of these discussions are presented below.

#### Results for stage 4

We summarize the findings from these discussions within 3 main themes: 1) data, 2) methods, and 3) presentation format. We develop 2 alternative display options to exemplify additional ways of presenting the findings.

#### Data

The prevailing view was that the main results were plausible (e.g., those for people aged >75 y) but that some should be interpreted with caution. In particular, the lack of granular information with respect to the severity of some conditions, for example, the stage of the cancer, whether diabetes was type 1 or type 2, and whether hypertension was controlled, must be acknowledged. Some comorbidities might be imperfectly captured in the data (e.g., due to changes in coding practices). Hence, the clinical co-investigators put the findings through a critical lens to help sift which were helpful for informing decision making and which were not.

#### Methods

Some of the clinicians wanted clarification as to which stage of LASSO the subgroups were selected (see stage 2). Similarly, it could be helpful to understand what determines the width of the confidence intervals (e.g., similar sample sizes may have CIs of different widths, for example, due to difference in the variability of individual-level effect estimates). They felt that forcing specific covariates into the models was helpful in generating subgroups of particular interest to them but that it was also useful for the ML to consider that there may be effect modification according to many other covariates alone and in combination.

#### Data display format

**Figure 2:**
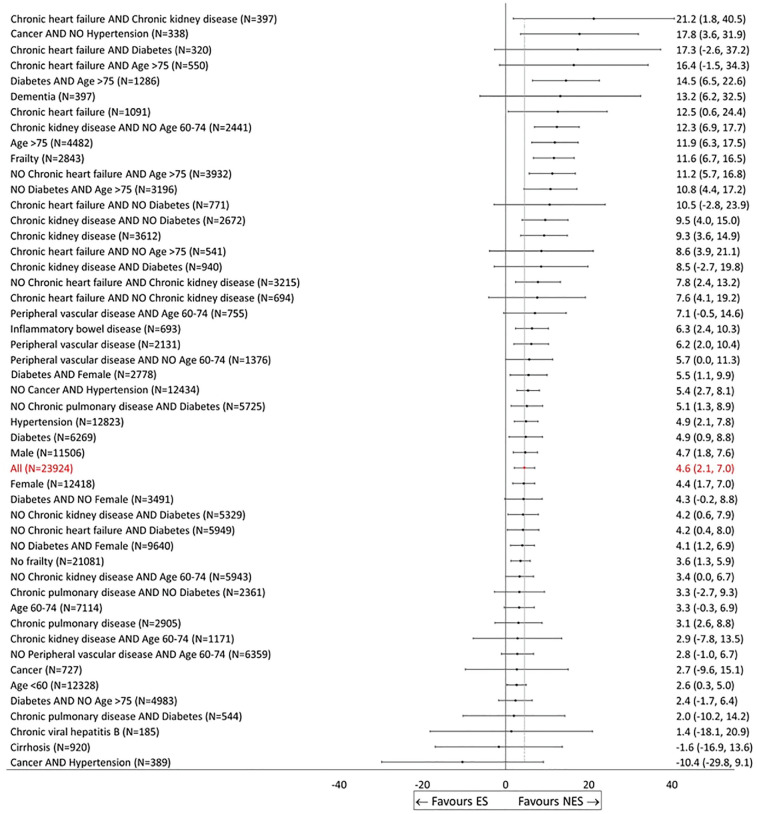
Effectiveness of nonemergency surgery (NES) strategies versus emergency surgery (ES) on days alive and out of hospital up to 90 d by subgroup (display option, No. 1).

The clinical co-investigators expressed different preferences about the grouping and the ordering of the subgroups. For instance, some preferred seeing all the results in 1 graph, whereas others found it challenging to interpret findings from this number of subgroups and preferred forest plots with fewer results. In Figure 2, patients could belong to different subgroups, which could complicate the interpretation. One way of partially addressing this would be to group results according to combinations of patient characteristics, such as age and frailty or individual comorbidities. Some of the clinical investigators expressed a preference for visualizing the results ordered by effect size (as in Figure 2), whereas others would prefer alternative ordering, for example, to be according to groupings/clusters of comorbidities. They also requested that the figure reported numeric values for the effect sizes and confidence intervals, sample sizes for the subgroups, and NES/ES rates for each subgroup.

The clinical co-investigators recognized that the presentation exemplified by Figure 2 was most helpful for informing those decisions when the effect for a single subgroup was of prime interest. A downside of this ordering of results by magnitude is that groups that are more closely related (e.g., age groups) are not necessarily displayed together, which can impede comparisons. Encouraged by the feedback from the clinical co-investigators, we now consider 2 complementary ways of presenting results.

A more flexible approach would allow the user to choose the covariates by which to stratify the sample. Inevitably there is a precision-interpretability tradeoff when choosing the level of aggregation at which to present effect estimates. While disaggregated subgroups are more targeted for an individual patient in that subgroup, with very refined groups it is difficult to identify clinically relevant recommendations. At the link https://github.com/silviamoler/ESORT-M, we offer a dynamic forest plot by which the user can visualize estimates of relative effectiveness for different combinations of patient subgroups to help inform the decision of prime interest. However, some caution is required here since the estimated effects have uncertainty associated with them that must be appropriately conveyed, and considering many groups may lead to discoveries that fail to generalize, an issue akin to multiple testing bias.

## Discussion

We present a 4-stage approach for integrating clinical judgment into a ML model (LASSO) for informing decision making. This approach to harnessing clinical judgment can help address concerns with fully data-driven approaches and increase trust in ML to help improve decision making. The 4 stages are model selection using 1) clinical judgment and 2) ML methods, 3) estimation of subgroup effects, and 4) selecting and reporting of results for decision making. We illustrate this approach in undertaking a new evaluation of NES strategies for acute appendicitis in patients with MLTCs. Here, the key role for clinical judgment is in co-designing the generation of evidence to prespecify potential drivers of heterogeneity in effects (modifiers) and help refine the reporting of results. However, the broad approach described of drawing on clinical judgment to inform the design of ML models and the presentation of results is applicable to other settings.

This article makes 3 important contributions to the literature. First, we add to efforts to increase trust and foster the appropriate adoption of ML in clinical decision making, which requires enhancing clinicians’ and in many settings patients’ understanding the purpose and limitations of ML models. Using expert judgment plays a key role in building users’ trust in the study’s findings.^23^ We complement this rapidly growing literature by developing a transparent approach for integrating clinical judgment into the use of ML, in this case LASSO. We anticipate that a similar principled approach may be beneficial in building trust in other ML algorithms, such as those relying on random forests. For methods that implicitly rely on complex combinations of covariates, it may be helpful to assess which variables drive predictions, for instance, using SHAP (SHapley Additive exPlanations) values,^67^ and to consider how these correspond to those identified by clinicians as being important. Thus, our work seeks to further a broader research agenda on improving the transparency of ML approaches.

Our study also complements the related literature of “explainable ML,” which aims to help users understand the predictions of ML models. In addition to methods based on partial dependence plots,^68^ which allow researchers to investigate the contribution of variables to a model’s output, the use of “model fact” labels or “model cards” to provide clear instructions on how the evidence of the model should be used might be helpful.^69,70^ These approaches may be helpful in improving assessments of treatment effect heterogeneity. Tools have been previously developed to plot output from ML prediction models and their performance (e.g., accuracy, recall, etc.) in an interactive way.^71^ We emphasize the key role of graphical tools in the comprehension and translation of ML output. Our tool allows users to plot the evidence in ways deemed most useful by them, according to users’ needs.

Second, this is the first study to formally evaluate heterogeneity in the clinical effectiveness of NES versus ES for acute appendicitis patients with MLTCs. Antibiotic therapy may be the best treatment option for patients with serious comorbidities given the high risks associated with surgery,^29^ but there is little empirical evidence. We find that NES, including antibiotic therapy, leads to better clinical outcomes than ES for MLTC patients overall and particularly for those who are older, frail, or have chronic heart failure, chronic kidney disease, or diabetes. Although, for simplicity this article focused on the main clinical outcome (DAOH at 90 d), our precedent research suggests that this is strongly predictive of cost-effectiveness.^31,32^

Third, the study contributes to the literature on data-adaptive approaches for IV estimation. We extend PDS for use with LIV methods building on Belloni et al.^48^ Similar approaches have been adopted in the context of binary IVs^72,73^ but had not yet been considered for IVs that have multiple values or are continuous.

The study has several strengths. First, we used large routine data that covered the full population of interest in England, for which rich clinical and sociodemographic information could be retrieved. Second, we adopted a co-production approach that recognized the dual role of the clinical co-investigators as both researchers and users of research evidence in the context of decision making in clinical practice. Third, we applied robust causal inference methods described in Moler-Zapata et al.^31^ for addressing the likely bias due to confounding and heterogeneity.

**Display Option 2:** Plot of Main Covariate Effects

**Figure 3:**
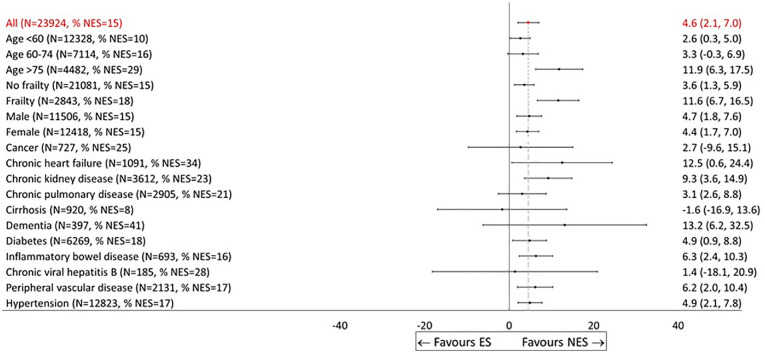
Effectiveness of nonemergency surgery (NES) strategies versus emergency surgery (ES) on days alive and out of hospital (DAOH) up to 90 d by subgroup (display option, No. 2). Only subgroups for main effects are reported in this figure. Subgroups defined by age, sex, and frailty levels appear as consecutive rows. Labels have been modified to include the percentage of patients who received NES in that subgroup.

The patient’s age and frailty level are factors that clinicians often take into consideration when making decisions about allocation to ES or NES. Figure 3 allows the reader to see how effectiveness varies across age groups or frailty levels. It also reveals differences in relative effectiveness according to whether patients had chronic heart failure, chronic kidney disease, and dementia, albeit with wide statistical uncertainty for some of these subgroups.

**Display Option 3:** Plot of Specific Patient Characteristics

**Figure 4:**
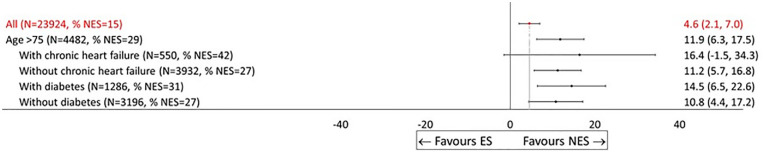
Effectiveness of nonemergency surgery (NES) strategies versus emergency surgery (ES) on days alive and out of hospital (DAOH) up to 90 d by subgroup (display option, No. 3). Labels have been modified to clearly reflect to which subgroup each row refers. Indentation is used to indicate whether the subgroup is defined by individual comorbidity or an interaction.

Some of the clinicians requested that results were presented to focus on specific subgroups of prime interest. Figure 4 takes this approach in showing a forest plot with treatment effects solely for patients aged 75 y or older. The average gains from NES versus ES are relatively large and precisely estimated with relatively little modification according to whether the patient has chronic heart failure or diabetes. Similar plots could be produced for those subgroups who are frail or who have chronic heart failure or dementia.

This study had a number of limitations. First, as is the case with many routinely collected administrative datasets, these data lacked fine-grade coding on measures of disease severity (e.g., cancer progression, diabetes type) and other patient characteristics (e.g., body mass index), whereas in practice, clinicians would have access to this more granular data. Therefore, decision making at the bedside will continue to require careful clinical judgment. Improved data linkage and/or triangulating evidence from multiple data sources could help address this shortcoming. Second, this study did not explore other issues with ML such as the risk of algorithmic bias^iv^ or the “fairness” of decisions.^15,74^ Third, the study drew on clinical judgment from 6 clinical co-investigators and did not extend the elicitation of judgment to a larger sample of external clinicians. The approach we used benefited from our clinical investigators’ familiarity with the general research question and a level of trust that allowed for a pragmatic and less structured approach to eliciting their clinical judgment. However, their views may differ from those of a larger external sample of clinicians. Lastly, in interpreting the results, given the number of comparisons undertaken, a clear concern is multiple testing bias leading to false discoveries. This concern coupled with the wide uncertainty surrounding the effects for individual subgroups limits the extent to which the study provides a strong basis for definitively recommending differential treatment decisions by subgroup. One could apply post hoc corrections to account for the number of tests undertaken, albeit some such corrections (e.g., Bonferroni^75^) can be overly conservative. Here, a useful output from this study is in raising new hypotheses for future research, in proposing more nuanced subgroup combinations that may modify the relative effectiveness of NES versus ES, so we do not do so here.^76^

This study identifies areas for future research. The setting exemplifies the potential to integrate clinical judgment into ML and could be generalized in different ways. For example, the clinical input could have adopted more formal approaches such as a Delphi Panel or survey of a larger sample of external clinicians for achieved expert consensus. In settings in which selection on observables is plausible, other ML approaches could have been considered such as meta-learners^77^ or causal forests.^78^ Other variable selection methods could be considered such as the “group LASSO,” which allows predefined groups of covariates to jointly be selected into or out of a model, potentially leading to a more interpretable model.^79^ One could explore other data-driven approaches for identifying subgroups (see, e.g., Su et al.,^10^ Lipkovich et al.,^11^ Foster et al.,^59^ Loh et al.,^60^ Hapfelmeier et al.,^61^ Dwivedi et al.,^62^ and Dusseldorp and Van Mechelen^63^), which split the data by characteristics predictive of expected treatment benefit. Since many of these are tree based, they result in mutually exclusive subgroups, which may be more interpretable. Our approach could be contrasted with other alternatives, such as using ML and clinical judgment to develop a single risk score to predict relative effectiveness and target interventions.

Our approach can be generalized in several ways. Building on recent work by Rodrigues et al.,^80^ one could use expert judgment in stage 1 to design a directed acyclic graph (DAG) representing the causal relationships between variables. This would allow us to identify the set of covariates for adjustment that ensures both that 1) the risk of confounding is minimized and 2) no post-treatment variables such as colliders (i.e., variables influence by treatment and outcomes) or mediators (intermediate variable that lies on the causal pathway) are controlled for either by analysis or in the design stage through sample selection. Evaluating the implications of alternative assumptions about the DAG could be assessed in sensitivity analyses. In Supplementary Table S2, we show that treatment effects estimated for subgroups identified in stage 2 (see Table 3) do not vary when 1) expert judgment is not used for identification of effect modifiers (fully data driven; column 4) and 2) only expert judgment is used (column 5). However, this does not constitute a formal test for bias, and results could be subject to the problems described in section 1 with fully data-driven approaches.

While our approach drew on judgment from clinical decision makers, which was appropriate in this emergency setting, for chronic conditions, it may be more appropriate to include judgment from patients and the public in combination with ML to inform models of shared decision making and indeed the use of decision aids, such as QRISK, which is used to inform initiation of statins for the primary prevention of cardiovascular disease in the United Kingdom.^81,82^ Setting up patient and public involvement (PPI) panels for deciding how evidence should be presented to a lay population to facilitate shared decision-making processes could be helpful. These panels should recognize that patients may be less familiar with some concepts discussed, and therefore, PPI panel participants would need to be offered simple, clear materials and training to ensure these are accessible. The ESORT project successfully set up PPI panels to discuss aspects of the study design, including endpoints.^83^ Alternative approaches to preference elicitation, including discrete choice experiments, to explore which graphics could be more visually accessible, appealing, and easy to interpret for patients would need to be considered.

In conclusion, our approach combining ML methods, in this case LASSO, with clinical judgment improves evidence generation for clinical decision making by increasing transparency, interpretability, and trust.

## Supplemental Material

sj-docx-1-mdm-10.1177_0272989X241289336 – Supplemental material for An Approach for Combining Clinical Judgment with Machine Learning to Inform Medical Decision Making: Analysis of Nonemergency Surgery Strategies for Acute Appendicitis in Patients with Multiple Long-Term Conditions Supplemental material, sj-docx-1-mdm-10.1177_0272989X241289336 for An Approach for Combining Clinical Judgment with Machine Learning to Inform Medical Decision Making: Analysis of Nonemergency Surgery Strategies for Acute Appendicitis in Patients with Multiple Long-Term Conditions by S. Moler-Zapata, A. Hutchings, R. Grieve, R. Hinchliffe, N. Smart, S. R. Moonesinghe, G. Bellingan, R. Vohra, S. Moug and S. O’Neill in Medical Decision Making

## Notes

i. Tacit knowledge (or “know-how”) is context-specific knowledge that is acquired through previous experience and is hard to transmit. It is different from “explicit” or “codified” knowledge (i.e., knowledge that can be acquired through research).^26^

ii. While in principle we could include the variables identified by clinicians among the variables for possible selection and allow the LASSO algorithm to assess whether they warrant inclusion, here we force their inclusion, which would increase efficiency if these variables are truly influential.

iii. We follow previous recommendations, including those from National Institute for Health and Care Excellence methods guidance, and do not consider the patient’s ethnicity or IMD in stage 1 or 2, as it would not generally be appropriate to issue differential treatment recommendations according to ethnicity or deprivation.^58^

iv. Algorithmic bias occurs when data-driven approaches replicate and even amplify human biases present in the data.
