# Supplementary material for: An Approach for Combining Clinical Judgment with Machine Learning to Inform Medical Decision Making: Analysis of Nonemergency Surgery Strategies for Acute Appendicitis in Patients with Multiple Long-Term Conditions
Source: Med Decis Making. 2024 Oct 23;44(8):944–60. doi: 10.1177/0272989X241289336 (PMC11542320; doi:10.1177/0272989X241289336)
Supplement: sj-docx-1-mdm-10.1177_0272989X241289336 – Supplemental material for An Approach for Combining Clinical Judgment with Machine Learning to Inform Medical Decision Making: Analysis of Nonemergency Surgery Strategies for Acute Appendicitis in Patients with Multiple Long-Term Conditions [file sj-docx-1-mdm-10.1177_0272989X241289336.docx]

**Supplementary materials for:**

**“An approach for combining clinical judgment with machine learning to inform medical decision-making: analysis of non-emergency surgery strategies for acute appendicitis in patients with multiple long-term conditions”**

**Contents**

**Supplemental notes**

S1 The Least Absolute Shrinkage and Selection Operator (LASSO) approach

S2 Local Instrumental Variable (LIV) estimator

**References**

**Supplemental tables**

S1 Full list of covariates considered for inclusion (individual comorbidities and interactions)

S2 Estimates of effectiveness of NES versus ES on days alive and out of hospital up to 90 days by subgroup

**Supplemental notes**

**Section 1: The Least Absolute Shrinkage and Selection Operator (LASSO) approach**

LASSO aims to find the set of coefficients that minimises the sum-of-squared errors subject to a constraint on the sum of absolute values of coefficients. A penalty function is added to the typical OLS loss function, as follows:

$$Loss= \sum_{i=1}^{N} \left( Y_{i}-\sum_{j=1}^{J} \beta_{j}x_{ij} \right)^{2}+\lambda\sum_{j=1}^{J} |\beta_{j}|$$

where Y represents the dependent variable, in our case this is either the treatment or outcome variable depending on the context, $x_{ij}$ is the *j*^th^ of *J* covariates for individual *i* and $\beta_{j}$ is the corresponding coefficient. The tuning parameter, $\lambda$, determines the extent to which model complexity is penalised, with larger values resulting in more variables being excluded. Various approaches have been suggested for choosing $\lambda$ including cross-validation and ‘rigorous LASSO’ [1]. Below, we use the ‘rigorous LASSO’ approach which places a high priority on controlling overfitting in the presence of heteroskedastic, non-Gaussian, and cluster-dependent errors, thus often producing parsimonious models [2].

**Section 2: Local Instrumental Variable (LIV) estimator**

Overview of LIV

Heckman and Vytlacil [3–5] show that the LIV estimator can be used to estimate series of Marginal Treatment Effects (MTEs), that is the average effect for “marginal” patients, those for whom there was equipoise about the ES decision according to these measured characteristics (e.g. age, TTO), as well as those unmeasured in the data (e.g. physiology). The MTE is defined as

$$\Delta^{MTE}\left( x_{O},p \right)=\frac{\partial E(Y_{1}-Y_{0}|X_{O}=x_{O}, P\left( z,x_{O} \right)=p)}{\partial p}$$

Where $Y_{1}$ and $Y_{0}$ are the potential outcome under treatment and control respectively, $X_{O}$ are the included variables, *Z* is the instrumental variable and *p* is the propensity for treatment. For these marginal patients, the instrumental variable approach estimates treatment effects for patients for whom a small change (or nudge) in the TTO (the instrument), can ‘tip the balance’ towards ES, but does not change the level of any risk factors, including those that are not unmeasured. Comparing outcomes for patients defined according to small differences in the TTO therefore provides an estimate of the causal effect of ES versus NES for similar patients. For a given combination of observed covariates, different levels of the IV would imply different levels of unobserved confounding at which the individual would be in equipoise. Therefore, by repeating this contrast across different levels of TTO, the study can estimate treatment effects for sets of marginal patients with different combinations of confounders (e.g. frailty levels). The MTEs relate to patients in equipoise rather than to each individual in the sample. Basu [6] showed that MTEs can be used to derive person-centered treatment (PeT) effects for each individual in the sample by averaging the MTEs for a given level of X and Z over those values of unobserved variables (defined on the probability scale) that are compatible with that patient’s actual treatment assignment [6, 7]. PeT effects for each individual are obtained by averaging the treatment effects for those marginal patients who share the same observed characteristics and IV level, and who have estimated levels of unobserved confounders that are consistent with the observed ES decision for that individual. The typical implementation of this approach relies on parametric models where the control variables and effect modifiers are known a priori rather than discovered from the data [7]. The precedent studies by Moler-Zapata et al., [8] and Hutchings et al [9] applied this LIV approach to estimate the effects of Es versus NES for acute conditions.

A data-adaptive LIV estimator

Here, we extend the LIV approach of Basu [6, 7] to be data adaptive, by including the forced and selected variables from stages 1 and 2 using the following steps:

- Step 1: estimate the propensity for treatment as a function of the instrument (Z), the forced variables, and the selected effect modifiers, $X_{\boldsymbol{Selected}}$:

$P\left( D=1 \right|Z, X_{Forced},X_{Selected})=\Phi(\alpha_{0}+\alpha_{1}Z+X_{Forced}\alpha_{2}+X_{Selected}\alpha_{3}$)

- Step 2: store the estimated propensity scores (*ps*) and make sure that there exists coverage for both treatment arms across all values from 0 to 1 (rounded to 0.01); dropping observations with poor overlap.
- Step 3: estimate an outcome model (GLM here) on the forced and selected covariates, the (estimated) propensity score and interactions of the propensity score with the selected effect modifiers,

$$Y=\beta_{0} + X_{Forced}\beta_{1}+X_{Selected}\beta_{2}+\beta_{3}\hat{ps}+\beta_{4}\hat{ps}^{2}+ \left( \hat{ps}*X_{Selected} \right)\tau+\epsilon$$

- Step 4: take the derivative of the estimated outcome equation with respect to the estimated propensity score to obtain the MTE estimates.
- Step 5**:** use numerical integration to obtain PeT effects for each individual in the sample as described in Basu [6, 7].

**References**

[1] Belloni A, Chen D, Chernozhukov V, et al. Sparse Models and Methods for Optimal Instruments With an Application to Eminent Domain. *Econometrica* 2012; 80: 2369–2429.

[2] Ahrens A, Hansen CB, Schaffer ME. lassopack: Model selection and prediction with regularized regression in Stata. *Stata J* 2020; 20: 176–235.

[3] Heckman JJ, Vytlacil EJ. Local instrumental variables and latent variable models for identifying and bounding treatment effects. *Proc Natl Acad Sci U S A* 1999; 96: 4730–4734.

[4] Heckman JJ, Vytlacil EJ. Policy-Relevant Treatment Effects. *Am Econ Rev* 2001; 91: 107–111.

[5] Heckman JJ, Vytlacil E. Structural equations, treatment effects and econometric policy evaluation. *Econometrica* 2005; 73: 669–738.

[6] Basu A. Estimating person-centered treatment (PeT) effects using instrumental variables: an application to evaluating prostate cancer treatments. *J Appl Econom* 2014; 29: 671–691.

[7] Basu A. Person-centered treatment (PeT) effects: Individualized treatment effects using instrumental variables. *Stata J* 2015; 15: 397–410.

[8] Moler-Zapata S, Grieve R, Lugo-Palacios D, et al. Local instrumental variable methods to address confounding and heterogeneity when using electronic health records: an application to emergency surgery. *Med Decis Mak*; 0. Epub ahead of print 24 May 2022. DOI: 10.1177/0272989X221100799.

[9] Hutchings A, O’Neill S, Lugo‐Palacios D, et al. Effectiveness of emergency surgery for five common acute conditions: an instrumental variable analysis of a national routine database. *Anaesthesia* 2022; 77: 865–881.

**Supplemental Tables**

**Table S1: Full list of covariates considered for inclusion (individual comorbidities and interactions)**

| \| Alcohol misuse AND Age 60-74 \| Asthma AND Chronic pain \| \| --- \| --- \| \| Alcohol misuse AND Age >75 \| Asthma AND Chronic pulmonary disease \| \| Alcohol misuse AND Frailty level 2 \| Asthma AND Chronic viral hepatitis B \| \| Alcohol misuse AND Female \| Asthma AND Cirrhosis \| \| Alcohol misuse \| Asthma AND Dementia \| \| Alcohol misuse AND Asthma \| Asthma AND Depression \| \| Alcohol misuse AND Atrial fibrillation \| Asthma AND Diabetes \| \| Alcohol misuse AND Cancer \| Asthma AND Epilepsy \| \| Alcohol misuse AND Chronic heart failure \| Asthma AND Hypertension \| \| Alcohol misuse AND Chronic kidney disease \| Asthma AND Hypothyroidism \| \| Alcohol misuse AND Chronic pain \| Asthma AND Inflammatory bowel disease \| \| Alcohol misuse AND Chronic pulmonary disease \| Asthma AND Irritable bowel syndrome \| \| Alcohol misuse AND Chronic viral hepatitis B \| Asthma AND Multiple sclerosis \| \| Alcohol misuse AND Cirrhosis \| Asthma AND Myocardial infarction \| \| Alcohol misuse AND Dementia \| Asthma AND Parkinson's disease \| \| Alcohol misuse AND Depression \| Asthma AND Peptic ulcer disease \| \| Alcohol misuse AND Diabetes \| Asthma AND Peripheral vascular disease \| \| Alcohol misuse AND Epilepsy \| Asthma AND Psoriasis \| \| Alcohol misuse AND Hypertension \| Asthma AND Rheumatoid arthritis \| \| Alcohol misuse AND Hypothyroidism \| Asthma AND Schizophrenia \| \| Alcohol misuse AND Inflammatory bowel disease \| Asthma AND Severe constipation \| \| Alcohol misuse AND Irritable bowel syndrome \| Asthma AND Stroke or TIA \| \| Alcohol misuse AND Multiple sclerosis \| Atrial fibrillation AND Age 60-74 \| \| Alcohol misuse AND Myocardial infarction \| Atrial fibrillation AND Age >75 \| \| Alcohol misuse AND Parkinson's disease \| Atrial fibrillation AND Frailty level 2 \| \| Alcohol misuse AND Peptic ulcer disease \| Atrial fibrillation AND Female \| \| Alcohol misuse AND Peripheral vascular disease \| Atrial fibrillation \| \| Alcohol misuse AND Psoriasis \| Atrial fibrillation AND Cancer \| \| Alcohol misuse AND Rheumatoid arthritis \| Atrial fibrillation AND Chronic heart failure \| \| Alcohol misuse AND Schizophrenia \| Atrial fibrillation AND Chronic kidney disease \| \| Alcohol misuse AND Severe constipation \| Atrial fibrillation AND Chronic pain \| \| Alcohol misuse AND Stroke or TIA \| Atrial fibrillation AND Chronic pulmonary disease \| \| Asthma AND Age 60-74 \| Atrial fibrillation AND Chronic viral hepatitis B \| \| Asthma AND Age >75 \| Atrial fibrillation AND Cirrhosis \| \| Asthma AND Frailty level 2 \| Atrial fibrillation AND Dementia \| \| Asthma AND Female \| Atrial fibrillation AND Depression \| \| Asthma \| Atrial fibrillation AND Diabetes \| \| Asthma AND Atrial fibrillation \| Atrial fibrillation AND Epilepsy \| \| Asthma AND Cancer \| Atrial fibrillation AND Hypertension \| \| Asthma AND Chronic heart failure \| Atrial fibrillation AND Hypothyroidism \| \| Asthma AND Chronic kidney disease \| Atrial fibrillation AND Inflammatory bowel disease \| \| Atrial fibrillation AND Irritable bowel syndrome \| Chronic heart failure AND Dementia \| \| Atrial fibrillation AND Multiple sclerosis \| Chronic heart failure AND Depression \| \| Atrial fibrillation AND Myocardial infarction \| Chronic heart failure AND Diabetes \| \| Atrial fibrillation AND Parkinson's disease \| Chronic heart failure AND Epilepsy \| \| Atrial fibrillation AND Peptic ulcer disease \| Chronic heart failure AND Hypertension \| \| Atrial fibrillation AND Peripheral vascular disease \| Chronic heart failure AND Hypothyroidism \| \| Atrial fibrillation AND Psoriasis \| Chronic heart failure AND Inflammatory bowel disease \| \| Atrial fibrillation AND Rheumatoid arthritis \| Chronic heart failure AND Irritable bowel syndrome \| \| Atrial fibrillation AND Schizophrenia \| Chronic heart failure AND Multiple sclerosis \| \| Atrial fibrillation AND Severe constipation \| Chronic heart failure AND Myocardial infarction \| \| Atrial fibrillation AND Stroke or TIA \| Chronic heart failure AND Parkinson's disease \| \| Cancer AND Age 60-74 \| Chronic heart failure AND Peptic ulcer disease \| \| Cancer AND Age >75 \| Chronic heart failure AND Peripheral vascular disease \| \| Cancer AND Frailty level 2 \| Chronic heart failure AND Psoriasis \| \| Cancer AND Female \| Chronic heart failure AND Rheumatoid arthritis \| \| Cancer AND Chronic heart failure \| Chronic heart failure AND Schizophrenia \| \| Cancer AND Chronic kidney disease \| Chronic heart failure AND Severe constipation \| \| Cancer AND Chronic pain \| Chronic heart failure AND Stroke or TIA \| \| Cancer AND Chronic pulmonary disease \| Chronic kidney disease AND Age 60-74 \| \| Cancer AND Chronic viral hepatitis B \| Chronic kidney disease AND Age >75 \| \| Cancer AND Cirrhosis \| Chronic kidney disease AND Frailty level 2 \| \| Cancer AND Dementia \| Chronic kidney disease AND Female \| \| Cancer AND Depression \| Chronic kidney disease AND Chronic pain \| \| Cancer AND Diabetes \| Chronic kidney disease AND Chronic pulmonary disease \| \| Cancer AND Epilepsy \| Chronic kidney disease AND Chronic viral hepatitis B \| \| Cancer AND Hypertension \| Chronic kidney disease AND Cirrhosis \| \| Cancer AND Hypothyroidism \| Chronic kidney disease AND Dementia \| \| Cancer AND Inflammatory bowel disease \| Chronic kidney disease AND Depression \| \| Cancer AND Irritable bowel syndrome \| Chronic kidney disease AND Diabetes \| \| Cancer AND Multiple sclerosis \| Chronic kidney disease AND Epilepsy \| \| Cancer AND Myocardial infarction \| Chronic kidney disease AND Hypertension \| \| Cancer AND Parkinson's disease \| Chronic kidney disease AND Hypothyroidism \| \| Cancer AND Peptic ulcer disease \| Chronic kidney disease AND Inflammatory bowel disease \| \| Cancer AND Peripheral vascular disease \| Chronic kidney disease AND Irritable bowel syndrome \| \| Cancer AND Psoriasis \| Chronic kidney disease AND Multiple sclerosis \| \| Cancer AND Rheumatoid arthritis \| Chronic kidney disease AND Myocardial infarction \| \| Cancer AND Schizophrenia \| Chronic kidney disease AND Parkinson's disease \| \| Cancer AND Severe constipation \| Chronic kidney disease AND Peptic ulcer disease \| \| Cancer AND Stroke or TIA \| Chronic kidney disease AND Peripheral vascular disease \| \| Chronic heart failure AND Age 60-74 \| Chronic kidney disease AND Psoriasis \| \| Chronic heart failure AND Age >75 \| Chronic kidney disease AND Rheumatoid arthritis \| \| Chronic heart failure AND Frailty level 2 \| Chronic kidney disease AND Schizophrenia \| \| Chronic heart failure AND Female \| Chronic kidney disease AND Severe constipation \| \| Chronic heart failure AND Chronic kidney disease \| Chronic kidney disease AND Stroke or TIA \| \| Chronic heart failure AND Chronic pain \| Chronic pain AND Age 60-74 \| \| Chronic heart failure AND Chronic pulmonary disease \| Chronic pain AND Age >75 \| \| Chronic heart failure AND Chronic viral hepatitis B \| Chronic pain AND Frailty level 2 \| \| Chronic heart failure AND Cirrhosis \| Chronic pain AND Female \| \| Chronic pain \| Chronic viral hepatitis B AND Age 60-74 \| \| Chronic pain AND Chronic pulmonary disease \| Chronic viral hepatitis B AND Age >75 \| \| Chronic pain AND Chronic viral hepatitis B \| Chronic viral hepatitis B AND Frailty level 2 \| \| Chronic pain AND Cirrhosis \| Chronic viral hepatitis B AND Female \| \| Chronic pain AND Dementia \| Chronic viral hepatitis B \| \| Chronic pain AND Depression \| Chronic viral hepatitis B AND Cirrhosis \| \| Chronic pain AND Diabetes \| Chronic viral hepatitis B AND Dementia \| \| Chronic pain AND Epilepsy \| Chronic viral hepatitis B AND Depression \| \| Chronic pain AND Hypertension \| Chronic viral hepatitis B AND Diabetes \| \| Chronic pain AND Hypothyroidism \| Chronic viral hepatitis B AND Epilepsy \| \| Chronic pain AND Inflammatory bowel disease \| Chronic viral hepatitis B AND Hypertension \| \| Chronic pain AND Irritable bowel syndrome \| Chronic viral hepatitis B AND Hypothyroidism \| \| Chronic pain AND Multiple sclerosis \| Chronic viral hepatitis B AND Inflammatory bowel disease \| \| Chronic pain AND Myocardial infarction \| Chronic viral hepatitis B AND Irritable bowel syndrome \| \| Chronic pain AND Parkinson's disease \| Chronic viral hepatitis B AND Multiple sclerosis \| \| Chronic pain AND Peptic ulcer disease \| Chronic viral hepatitis B AND Myocardial infarction \| \| Chronic pain AND Peripheral vascular disease \| Chronic viral hepatitis B AND Parkinson's disease \| \| Chronic pain AND Psoriasis \| Chronic viral hepatitis B AND Peptic ulcer disease \| \| Chronic pain AND Rheumatoid arthritis \| Chronic viral hepatitis B AND Peripheral vascular disease \| \| Chronic pain AND Schizophrenia \| Chronic viral hepatitis B AND Psoriasis \| \| Chronic pain AND Severe constipation \| Chronic viral hepatitis B AND Rheumatoid arthritis \| \| Chronic pain AND Stroke or TIA \| Chronic viral hepatitis B AND Schizophrenia \| \| Chronic pulmonary disease AND Age 60-74 \| Chronic viral hepatitis B AND Severe constipation \| \| Chronic pulmonary disease AND Age >75 \| Chronic viral hepatitis B AND Stroke or TIA \| \| Chronic pulmonary disease AND Frailty level 2 \| Cirrhosis AND Age 60-74 \| \| Chronic pulmonary disease AND Female \| Cirrhosis AND Age >75 \| \| Chronic pulmonary disease AND Chronic viral hepatitis B \| Cirrhosis AND Frailty level 2 \| \| Chronic pulmonary disease AND Cirrhosis \| Cirrhosis AND Female \| \| Chronic pulmonary disease AND Dementia \| Cirrhosis AND Dementia \| \| Chronic pulmonary disease AND Depression \| Cirrhosis AND Depression \| \| Chronic pulmonary disease AND Diabetes \| Cirrhosis AND Diabetes \| \| Chronic pulmonary disease AND Epilepsy \| Cirrhosis AND Epilepsy \| \| Chronic pulmonary disease AND Hypertension \| Cirrhosis AND Hypertension \| \| Chronic pulmonary disease AND Hypothyroidism \| Cirrhosis AND Hypothyroidism \| \| Chronic pulmonary disease AND Inflammatory bowel disease \| Cirrhosis AND Inflammatory bowel disease \| \| Chronic pulmonary disease AND Irritable bowel syndrome \| Cirrhosis AND Irritable bowel syndrome \| \| Chronic pulmonary disease AND Multiple sclerosis \| Cirrhosis AND Multiple sclerosis \| \| Chronic pulmonary disease AND Myocardial infarction \| Cirrhosis AND Myocardial infarction \| \| Chronic pulmonary disease AND Parkinson's disease \| Cirrhosis AND Parkinson's disease \| \| Chronic pulmonary disease AND Peptic ulcer disease \| Cirrhosis AND Peptic ulcer disease \| \| Chronic pulmonary disease AND Peripheral vascular disease \| Cirrhosis AND Peripheral vascular disease \| \| Chronic pulmonary disease AND Psoriasis \| Cirrhosis AND Psoriasis \| \| Chronic pulmonary disease AND Rheumatoid arthritis \| Cirrhosis AND Rheumatoid arthritis \| \| Chronic pulmonary disease AND Schizophrenia \| Cirrhosis AND Schizophrenia \| \| Chronic pulmonary disease AND Severe constipation \| Cirrhosis AND Severe constipation \| \| Chronic pulmonary disease AND Stroke or TIA \| Cirrhosis AND Stroke or TIA \| \|  \|  \| \|  \|  \| \| Dementia AND Age 60-74 \| Diabetes AND Hypothyroidism \| \| Dementia AND Age >75 \| Diabetes AND Inflammatory bowel disease \| \| Dementia AND Frailty level 2 \| Diabetes AND Irritable bowel syndrome \| \| Dementia AND Female \| Diabetes AND Multiple sclerosis \| \| Dementia AND Depression \| Diabetes AND Myocardial infarction \| \| Dementia AND Diabetes \| Diabetes AND Parkinson's disease \| \| Dementia AND Epilepsy \| Diabetes AND Peptic ulcer disease \| \| Dementia AND Hypertension \| Diabetes AND Peripheral vascular disease \| \| Dementia AND Hypothyroidism \| Diabetes AND Psoriasis \| \| Dementia AND Inflammatory bowel disease \| Diabetes AND Rheumatoid arthritis \| \| Dementia AND Irritable bowel syndrome \| Diabetes AND Schizophrenia \| \| Dementia AND Multiple sclerosis \| Diabetes AND Severe constipation \| \| Dementia AND Myocardial infarction \| Diabetes AND Stroke or TIA \| \| Dementia AND Parkinson's disease \| Epilepsy AND Age 60-74 \| \| Dementia AND Peptic ulcer disease \| Epilepsy AND Age >75 \| \| Dementia AND Peripheral vascular disease \| Epilepsy AND Frailty level 2 \| \| Dementia AND Psoriasis \| Epilepsy AND Female \| \| Dementia AND Rheumatoid arthritis \| Epilepsy \| \| Dementia AND Schizophrenia \| Epilepsy AND Hypertension \| \| Dementia AND Severe constipation \| Epilepsy AND Hypothyroidism \| \| Dementia AND Stroke or TIA \| Epilepsy AND Inflammatory bowel disease \| \| Depression AND Age 60-74 \| Epilepsy AND Irritable bowel syndrome \| \| Depression AND Age >75 \| Epilepsy AND Multiple sclerosis \| \| Depression AND Frailty level 2 \| Epilepsy AND Myocardial infarction \| \| Depression AND Female \| Epilepsy AND Parkinson's disease \| \| Depression \| Epilepsy AND Peptic ulcer disease \| \| Depression AND Diabetes \| Epilepsy AND Peripheral vascular disease \| \| Depression AND Epilepsy \| Epilepsy AND Psoriasis \| \| Depression AND Hypertension \| Epilepsy AND Rheumatoid arthritis \| \| Depression AND Hypothyroidism \| Epilepsy AND Schizophrenia \| \| Depression AND Inflammatory bowel disease \| Epilepsy AND Severe constipation \| \| Depression AND Irritable bowel syndrome \| Epilepsy AND Stroke or TIA \| \| Depression AND Multiple sclerosis \| Hypertension AND Age 60-74 \| \| Depression AND Myocardial infarction \| Hypertension AND Age >75 \| \| Depression AND Parkinson's disease \| Hypertension AND Frailty level 2 \| \| Depression AND Peptic ulcer disease \| Hypertension AND Female \| \| Depression AND Peripheral vascular disease \| Hypertension \| \| Depression AND Psoriasis \| Hypertension AND Hypothyroidism \| \| Depression AND Rheumatoid arthritis \| Hypertension AND Inflammatory bowel disease \| \| Depression AND Schizophrenia \| Hypertension AND Irritable bowel syndrome \| \| Depression AND Severe constipation \| Hypertension AND Multiple sclerosis \| \| Depression AND Stroke or TIA \| Hypertension AND Myocardial infarction \| \| Diabetes AND Age 60-74 \| Hypertension AND Parkinson's disease \| \| Diabetes AND Age >75 \| Hypertension AND Peptic ulcer disease \| \| Diabetes AND Frailty level 2 \| Hypertension AND Peripheral vascular disease \| \| Diabetes AND Female \| Hypertension AND Psoriasis \| \| Diabetes AND Epilepsy \| Hypertension AND Rheumatoid arthritis \| \| Diabetes AND Hypertension \| Hypertension AND Schizophrenia \| \| Hypertension AND Severe constipation \| Irritable bowel syndrome AND Stroke or TIA \| \| Hypertension AND Stroke or TIA \| Multiple sclerosis AND Age 60-74 \| \| Hypothyroidism AND Age 60-74 \| Multiple sclerosis AND Age >75 \| \| Hypothyroidism AND Age >75 \| Multiple sclerosis AND Frailty level 2 \| \| Hypothyroidism AND Frailty level 2 \| Multiple sclerosis AND Female \| \| Hypothyroidism AND Female \| Multiple sclerosis \| \| Hypothyroidism \| Multiple sclerosis AND Myocardial infarction \| \| Hypothyroidism AND Inflammatory bowel disease \| Multiple sclerosis AND Parkinson's disease \| \| Hypothyroidism AND Irritable bowel syndrome \| Multiple sclerosis AND Peptic ulcer disease \| \| Hypothyroidism AND Multiple sclerosis \| Multiple sclerosis AND Peripheral vascular disease \| \| Hypothyroidism AND Myocardial infarction \| Multiple sclerosis AND Psoriasis \| \| Hypothyroidism AND Parkinson's disease \| Multiple sclerosis AND Rheumatoid arthritis \| \| Hypothyroidism AND Peptic ulcer disease \| Multiple sclerosis AND Schizophrenia \| \| Hypothyroidism AND Peripheral vascular disease \| Multiple sclerosis AND Severe constipation \| \| Hypothyroidism AND Psoriasis \| Multiple sclerosis AND Stroke or TIA \| \| Hypothyroidism AND Rheumatoid arthritis \| Myocardial infarction AND Age 60-74 \| \| Hypothyroidism AND Schizophrenia \| Myocardial infarction AND Age >75 \| \| Hypothyroidism AND Severe constipation \| Myocardial infarction AND Frailty level 2 \| \| Hypothyroidism AND Stroke or TIA \| Myocardial infarction AND Female \| \| Inflammatory bowel disease AND Age 60-74 \| Myocardial infarction \| \| Inflammatory bowel disease AND Age >75 \| Myocardial infarction AND Parkinson's disease \| \| Inflammatory bowel disease AND Frailty level 2 \| Myocardial infarction AND Peptic ulcer disease \| \| Inflammatory bowel disease AND Female \| Myocardial infarction AND Peripheral vascular disease \| \| Inflammatory bowel disease \| Myocardial infarction AND Psoriasis \| \| Inflammatory bowel disease AND Multiple sclerosis \| Myocardial infarction AND Rheumatoid arthritis \| \| Inflammatory bowel disease AND Myocardial infarction \| Myocardial infarction AND Schizophrenia \| \| Inflammatory bowel disease AND Parkinson's disease \| Myocardial infarction AND Severe constipation \| \| Inflammatory bowel disease AND Peptic ulcer disease \| Myocardial infarction AND Stroke or TIA \| \| Inflammatory bowel disease AND Peripheral vascular disease \| Parkinson's disease AND Age 60-74 \| \| Inflammatory bowel disease AND Psoriasis \| Parkinson's disease AND Age >75 \| \| Inflammatory bowel disease AND Rheumatoid arthritis \| Parkinson's disease AND Frailty level 2 \| \| Inflammatory bowel disease AND Schizophrenia \| Parkinson's disease AND Female \| \| Inflammatory bowel disease AND Severe constipation \| Parkinson's disease \| \| Inflammatory bowel disease AND Stroke or TIA \| Parkinson's disease AND Peptic ulcer disease \| \| Irritable bowel syndrome AND Age 60-74 \| Parkinson's disease AND Peripheral vascular disease \| \| Irritable bowel syndrome AND Age >75 \| Parkinson's disease AND Psoriasis \| \| Irritable bowel syndrome AND Frailty level 2 \| Parkinson's disease AND Rheumatoid arthritis \| \| Irritable bowel syndrome AND Female \| Parkinson's disease AND Schizophrenia \| \| Irritable bowel syndrome \| Parkinson's disease AND Severe constipation \| \| Irritable bowel syndrome AND Multiple sclerosis \| Parkinson's disease AND Stroke or TIA \| \| Irritable bowel syndrome AND Myocardial infarction \| Peptic ulcer disease AND Age 60-74 \| \| Irritable bowel syndrome AND Parkinson's disease \| Peptic ulcer disease AND Age >75 \| \| Irritable bowel syndrome AND Peptic ulcer disease \| Peptic ulcer disease AND Frailty level 2 \| \| Irritable bowel syndrome AND Peripheral vascular disease \| Peptic ulcer disease AND Female \| \| Irritable bowel syndrome AND Psoriasis \| Peptic ulcer disease \| \| Irritable bowel syndrome AND Rheumatoid arthritis \| Peptic ulcer disease AND Peripheral vascular disease \| \| Irritable bowel syndrome AND Schizophrenia \| Peptic ulcer disease AND Psoriasis \| \| Irritable bowel syndrome AND Severe constipation \| Peptic ulcer disease AND Rheumatoid arthritis \| \| Peptic ulcer disease AND Schizophrenia \|  \| \| Peptic ulcer disease AND Severe constipation \|  \| \| Peptic ulcer disease AND Stroke or TIA \|  \| \| Peripheral vascular disease AND Age 60-74 \|  \| \| Peripheral vascular disease AND Age >75 \|  \| \| Peripheral vascular disease AND Frailty level 2 \|  \| \| Peripheral vascular disease AND Female \|  \| \| Peripheral vascular disease \|  \| \| Peripheral vascular disease AND Psoriasis \|  \| \| Peripheral vascular disease AND Rheumatoid arthritis \|  \| \| Peripheral vascular disease AND Schizophrenia \|  \| \| Peripheral vascular disease AND Severe constipation \|  \| \| Peripheral vascular disease AND Stroke or TIA \|  \| \| Psoriasis AND Age 60-74 \|  \| \| Psoriasis AND Age >75 \|  \| \| Psoriasis AND Frailty level 2 \|  \| \| Psoriasis AND Female \|  \| \| Psoriasis \|  \| \| Psoriasis AND Rheumatoid arthritis \|  \| \| Psoriasis AND Schizophrenia \|  \| \| Psoriasis AND Severe constipation \|  \| \| Psoriasis AND Stroke or TIA \|  \| \| Rheumatoid arthritis AND Age 60-74 \|  \| \| Rheumatoid arthritis AND Age >75 \|  \| \| Rheumatoid arthritis AND Frailty level 2 \|  \| \| Rheumatoid arthritis AND Female \|  \| \| Rheumatoid arthritis \|  \| \| Rheumatoid arthritis AND Schizophrenia \|  \| \| Rheumatoid arthritis AND Severe constipation \|  \| \| Rheumatoid arthritis AND Stroke or TIA \|  \| \| Schizophrenia AND Age 60-74 \|  \| \| Schizophrenia AND Age >75 \|  \| \| Schizophrenia AND Frailty level 2 \|  \| \| Schizophrenia AND Female \|  \| \| Schizophrenia \|  \| \| Schizophrenia AND Severe constipation \|  \| \| Schizophrenia AND Stroke or TIA \|  \| \| Severe constipation AND Age 60-74 \|  \| \| Severe constipation AND Age >75 \|  \| \| Severe constipation AND Frailty level 2 \|  \| \| Severe constipation AND Female \|  \| \| Severe constipation \|  \| \| Severe constipation AND Stroke or TIA \|  \| \| Stroke or TIA AND Age 60-74 \|  \| \| Stroke or TIA AND Age >75 \|  \| \| Stroke or TIA AND Frailty level 2 \|  \| \| Stroke or TIA AND Female \|  \| \| Stroke or TIA \|  \| |
| --- | --- | --- | --- | --- | --- | --- | --- | --- | --- | --- | --- | --- | --- | --- | --- | --- | --- | --- | --- | --- | --- | --- | --- | --- | --- | --- | --- | --- | --- | --- | --- | --- | --- | --- | --- | --- | --- | --- | --- | --- | --- | --- | --- | --- | --- | --- | --- | --- | --- | --- | --- | --- | --- | --- | --- | --- | --- | --- | --- | --- | --- | --- | --- | --- | --- | --- | --- | --- | --- | --- | --- | --- | --- | --- | --- | --- | --- | --- | --- | --- | --- | --- | --- | --- | --- | --- | --- | --- | --- | --- | --- | --- | --- | --- | --- | --- | --- | --- | --- | --- | --- | --- | --- | --- | --- | --- | --- | --- | --- | --- | --- | --- | --- | --- | --- | --- | --- | --- | --- | --- | --- | --- | --- | --- | --- | --- | --- | --- | --- | --- | --- | --- | --- | --- | --- | --- | --- | --- | --- | --- | --- | --- | --- | --- | --- | --- | --- | --- | --- | --- | --- | --- | --- | --- | --- | --- | --- | --- | --- | --- | --- | --- | --- | --- | --- | --- | --- | --- | --- | --- | --- | --- | --- | --- | --- | --- | --- | --- | --- | --- | --- | --- | --- | --- | --- | --- | --- | --- | --- | --- | --- | --- | --- | --- | --- | --- | --- | --- | --- | --- | --- | --- | --- | --- | --- | --- | --- | --- | --- | --- | --- | --- | --- | --- | --- | --- | --- | --- | --- | --- | --- | --- | --- | --- | --- | --- | --- | --- | --- | --- | --- | --- | --- | --- | --- | --- | --- | --- | --- | --- | --- | --- | --- | --- | --- | --- | --- | --- | --- | --- | --- | --- | --- | --- | --- | --- | --- | --- | --- | --- | --- | --- | --- | --- | --- | --- | --- | --- | --- | --- | --- | --- | --- | --- | --- | --- | --- | --- | --- | --- | --- | --- | --- | --- | --- | --- | --- | --- | --- | --- | --- | --- | --- | --- | --- | --- | --- | --- | --- | --- | --- | --- | --- | --- | --- | --- | --- | --- | --- | --- | --- | --- | --- | --- | --- | --- | --- | --- | --- | --- | --- | --- | --- | --- | --- | --- | --- | --- | --- | --- | --- | --- | --- | --- | --- | --- | --- | --- | --- | --- | --- | --- | --- | --- | --- | --- | --- | --- | --- | --- | --- | --- | --- | --- | --- | --- | --- | --- | --- | --- | --- | --- | --- | --- | --- | --- | --- | --- | --- | --- | --- | --- | --- | --- | --- | --- | --- | --- | --- | --- | --- | --- | --- | --- | --- | --- | --- | --- | --- | --- | --- | --- | --- | --- | --- | --- | --- | --- | --- | --- | --- | --- | --- | --- | --- | --- | --- | --- | --- | --- | --- | --- | --- | --- | --- | --- | --- | --- | --- | --- | --- | --- | --- | --- | --- | --- | --- | --- | --- | --- | --- | --- | --- | --- | --- | --- | --- | --- | --- | --- | --- | --- | --- | --- | --- | --- | --- | --- | --- | --- | --- | --- | --- | --- | --- | --- | --- | --- | --- | --- | --- | --- | --- | --- | --- | --- | --- | --- | --- | --- | --- | --- | --- | --- | --- | --- | --- | --- | --- | --- | --- | --- | --- | --- | --- | --- | --- | --- | --- | --- | --- | --- | --- | --- | --- | --- | --- | --- | --- | --- | --- | --- | --- | --- | --- | --- | --- | --- | --- | --- | --- | --- | --- | --- | --- | --- | --- | --- | --- | --- | --- | --- | --- | --- | --- | --- | --- | --- | --- | --- | --- | --- | --- | --- | --- | --- | --- | --- | --- | --- | --- | --- | --- | --- | --- | --- | --- | --- | --- | --- | --- | --- | --- | --- | --- | --- | --- | --- | --- | --- | --- | --- |

**Table S2: Estimates of effectiveness of NES versus ES on days alive and out of hospital up to 90 days by subgroup**

| **Subgroups** | **N** | **Mean difference (95% CI)** | | |
| --- | --- | --- | --- | --- |
|  |  | **Base case** | **Fully data-driven** | **Fully clinician-driven** |
| Overall | 23924 | -4.6 (-7.0, -2.1) | -4.5 (-6.8, -2.3) | -4.6 (-6.7, -2.4) |
| Age: <60 | 12328 | -2.6 (-5.0, -0.3) | -2.8 (-5.2, -0.5) | -3.1 (-5.4, -0.8) |
| Age: 60-74 | 7114 | -3.3 (-6.9, 0.3) | -2.9 (-6.9, 1.2) | -3.4 (-6.7, 0.0) |
| Age: >75 | 4482 | -11.9 (-17.5, -6.3) | -11.9 (-17.1, -6.7) | -10.5 (-15.5, -5.6) |
| Frailty level 0 | 21081 | -3.6 (-5.9, -1.3) | -3.6 (-5.8, -1.5) | -3.6 (-5.7, -1.6) |
| Frailty level 2 | 2843 | -11.6 (-16.5, -6.7) | -11.3 (-16.4, -6.3) | -11.6 (-16.2, -7.0) |
| Male | 11506 | -4.7 (-7.6, -1.8) | -4.5 (-7.1, -1.9) | -4.5 (-7.5, -1.5) |
| Female | 12418 | -4.4 (-7.0, -1.7) | -4.6 (-7.0, -2.1) | -4.6 (-6.6, -2.5) |
| Cancer* | 727 | -2.7 (-15.1, 9.6) | -5.0 (-15.8, 5.7) | -4.8 (-16.5, 6.9) |
| Chronic heart failure* | 1091 | -12.5 (-24.4, -0.6) | -12.1 (-24.6, 0.4) | -4.6 (-13.9, 4.7) |
| Chronic kidney disease* | 3612 | -9.3 (-14.9, -3.6) | -8.9 (-14.5, -3.3) | -8.7 (-13.3, -4.0) |
| Chronic pulmonary disease* | 2905 | -3.1 (-8.8, 2.6) | -3.6 (-9.5, 2.4) | -2.4 (-8.0, 3.2) |
| Cirrhosis* | 920 | 1.6 (-13.6, 16.9) | 2.0 (-10.1, 14.2) | 0.8 (-10.3, 11.8) |
| Dementia* | 397 | -13.2 (-32.5, 6.2) | -14.0 (-30.5, 2.5) | -16.5 (-35.1, 2.1) |
| Diabetes* | 6269 | -4.9 (-8.8, -0.9) | -4.7 (-8.6, -0.7) | -4.8 (-8.1, -1.5) |
| Inflammatory bowel disease* | 693 | -6.3 (-10.3, -2.4) | -3.3 (-5.3, -1.3) | -7.2 (-10.5, -3.8) |
| Chronic viral hepatitis B | 185 | -1.4 (-20.9, 18.1) | -1.3 (-19.0, 16.5) | -4.4 (-7.6, -1.2) |
| Age: >75 AND chronic heart failure | 550 | -16.4 (-34.3, 1.5) | -16.0 (-36.0, 4.1) | -8.9 (-20.0, 2.1) |
| Age: 60-74 AND chronic kidney disease | 1171 | -2.9 (-13.5, 7.8) | -2.2 (-13.5, 9.2) | -5.7 (-11.8, 0.5) |
| Age: >75 AND diabetes | 1286 | -14.5 (-22.6, -6.5) | -14.8 (-23.9, -5.7) | -10.6 (-16.2, -5.1) |
| Female AND diabetes | 2778 | -5.5 (-9.9, -1.1) | -5.6 (-9.7, -1.5) | -4.9 (-8.2, -1.6) |
| Diabetes AND chronic heart failure | 320 | -17.3 (-37.2, 2.6) | -16.4 (-35.7, 2.9) | -5.0 (-15.2, 5.3) |
| Diabetes AND chronic kidney disease | 940 | -8.5 (-19.8, 2.7) | -8.1 (-18.0, 1.7) | -9.1 (-15.2, -2.9) |
| Diabetes AND chronic pulmonary disease | 544 | -2.0 (-14.2, 10.2) | -2.8 (-15.2, 9.7) | -2.0 (-9.3, 5.2) |
| Age: 60-74 AND peripheral vascular disease | 755 | -7.1 (-14.6, 0.5) | -3.7 (-9.3, 1.9) | -3.4 (-6.9, 0.1) |
| Cancer AND hypertension | 389 | 10.4 (-9.1, 29.8) | -5.3 (-16.2, 5.7) | -4.9 (-16.9, 7.0) |
| Chronic kidney disease AND chronic heart failure | 397 | -21.2 (-40.5, -1.8) | -20.1 (-40.9, 0.7) | -7.4 (-19.3, 4.6) |
| Peripheral vascular disease | 2131 | -6.2 (-10.4, -2.0) | -6.2 (-10.9, -1.5) | -5.1 (-7.5, -2.7) |
| Hypertension | 12823 | -4.9 (-7.8, -2.1) | -5.2 (-7.6, -2.7) | -5.3 (-7.6, -3.0) |
| Chronic heart failure AND NO Age: >75 | 541 | -8.6 (-21.1, 3.9) | -8.2 (-21.2, 4.8) | -0.2 (-9.6, 9.3) |
| Age: >75 AND NO chronic heart failure | 3932 | -11.2 (-16.8, -5.7) | -11.4 (-16.2, -6.5) | -10.7 (-15.7, -5.7) |
| Chronic kidney disease AND NO Age: 60-74 | 2441 | -12.3 (-17.7, -6.9) | -12.2 (-17.7, -6.6) | -10.1 (-14.9, -5.4) |
| Age: 60-74 AND NO chronic kidney disease | 5943 | -3.4 (-6.7, -0.0) | -3.0 (-6.8, 0.9) | -2.9 (-6.0, 0.2) |
| Diabetes AND NO Age: >75 | 4983 | -2.4 (-6.4, 1.7) | -2.1 (-6.1, 2.0) | -3.3 (-6.7, 0.1) |
| Age: >75 AND NO diabetes | 3196 | -10.8 (-17.2, -4.4) | -10.7 (-16.7, -4.8) | -10.5 (-15.7, -5.2) |
| Diabetes AND NO female | 3491 | -4.3 (-8.8, 0.2) | -3.9 (-8.7, 0.9) | -4.7 (-8.6, -0.9) |
| Female AND NO diabetes | 9640 | -4.1 (-6.9, -1.2) | -4.2 (-6.8, -1.7) | -4.5 (-6.4, -2.5) |
| Chronic heart failure AND NO Diabetes | 771 | -10.5 (-23.9, 2.8) | -10.3 (-24.2, 3.6) | -4.4 (-14.0, 5.1) |
| Diabetes AND no chronic heart failure | 5949 | -4.2 (-8.0, -0.4) | -4.0 (-7.9, -0.2) | -4.8 (-8.1, -1.6) |
| Chronic kidney disease AND NO Diabetes | 2672 | -9.5 (-15.0, -4.0) | -9.2 (-15.4, -3.0) | -8.5 (-13.1, -4.0) |
| Diabetes AND no chronic kidney disease | 5329 | -4.2 (-7.9, -0.6) | -4.1 (-7.7, -0.4) | -4.1 (-7.2, -1.0) |
| Chronic pulmonary disease AND NO Diabetes | 2361 | -3.3 (-9.3, 2.7) | -3.7 (-10.3, 2.8) | -2.5 (-8.0, 3.1) |
| Diabetes AND no chronic pulmonary disease | 5725 | -5.1 (-8.9, -1.3) | -4.9 (-8.6, -1.1) | -5.1 (-8.2, -1.9) |
| Peripheral vascular disease AND NO Age: 60-74 | 1376 | -5.7 (-11.3, -0.0) | -7.6 (-12.5, -2.6) | -6.1 (-8.7, -3.5) |
| Age: 60-74 AND no peripheral vascular disease | 6359 | -2.8 (-6.7, 1.0) | -2.8 (-6.8, 1.3) | -3.4 (-6.7, 0.0) |
| Cancer AND NO hypertension | 338 | -17.8 (-31.9, -3.6) | -4.8 (-15.5, 5.9) | -4.7 (-16.3, 6.9) |
| Hypertension and NO cancer | 12434 | -5.4 (-8.1, -2.7) | -5.2 (-7.6, -2.7) | -5.3 (-7.6, -3.0) |
| Chronic heart failure AND no chronic kidney disease | 694 | -7.6 (-19.2, 4.1) | -7.5 (-19.3, 4.2) | -3.0 (-11.7, 5.7) |
| Chronic kidney disease AND no chronic heart failure | 3215 | -7.8 (-13.2, -2.4) | -7.5 (-13.0, -2.0) | -8.8 (-13.4, -4.3) |

Estimates of effectiveness for subgroups defined by covariates and covariate interactions identified as effect modifiers in the base case analysis were obtained following: i) a fully data-driven approach to effect modifier selection (column 4), and an approach where effect modifiers were selected clinical (column 5). In addition to covariates identified by LASSO (column 4) and the clinicians (column 5), models were adjusted for covariates listed on page 11. Results following the standpoints of the base case analysis are reported in column 3.

* Variable was identified by clinicians as covariate that must be included in the model and considered as effect modifiers.

ES: Emergency Surgery, NES: non-emergency surgery.
